# Correlation Between Enrollment of Students in Mentoring Program and Their Academic Achievements: A Cross-Sectional Study

**DOI:** 10.7759/cureus.19477

**Published:** 2021-11-11

**Authors:** Hussein Abdellatif, Mithaq Al-Balushi

**Affiliations:** 1 Department of Human and Clinical Anatomy, Sultan Qaboos University, College of Medicine and Health Sciences, Muscat, OMN; 2 Anatomy and Embryology Department, University of Mansoura, Faculty of Medicine, Mansoura, EGY; 3 Human and Clinical Anatomy, Sultan Qaboos University, College of Medicine and Health Sciences, Muscat, OMN

**Keywords:** cumulative grade point average (cgpa), academic success, medical education, medical students, mentoring program

## Abstract

Introduction

Mentoring is a process in which a mentor guides his mentee to achieve specific academic goals and an array of objectives. We conducted this study to detect the correlation between the active participation of the students in the mentoring program and their academic achievements.

Methods

This is a comparative cross-sectional study. The data were collected through an online questionnaire. One hundred participants were enrolled randomly in the study. The data included the number of meetings between the mentor and students and their cumulative grade point average (CGPA).

Results

The response rate was 83.3% (100 students). Fifty percent (n = 50) of the respondents had never met with their mentors while the other 50% (n = 50) had met with their mentors at least once in a semester. For this group, overall, positive response rates regarding the value and effectiveness of the mentoring program exceeded 78%. The correlation between participation in the mentoring program and the academic achievements of students was calculated (R^2^ was 0.007, *p-value* = 0.757).

Conclusions

This study demonstrated a non-significant correlation between the degree of involvement in the mentoring program and students’ overall academic achievements as students from both sections. Those who were enrolled in the program, and those students who were not, still achieved high scores.

## Introduction

A mentoring program is a process in which an experienced, wise, empathetic, and committed individual, known as the mentor, takes upon himself/herself the role of guiding another individual, known as a mentee, to achieve his or her specific academic goals and an array of objectives [1- 2]. These mentoring programs have become prominent tools in education and are considered a crucial step that guides the students in achieving their professional success, along with enhancing their personal growth. Therefore, effective mentoring programs enable the mentees to set a high level of expectations of their own career progress [3].

The aim of the mentoring program primarily depends upon the needs and desires of the mentees, where it is designed to help students to achieve their set of goals along their academic journey [4]. These nurturing, supportive, and intentional mentoring programs aid students, especially first-year undergraduates embarking on a professional course, to adapt to the new study atmosphere and enhance their sense of identity and belonging to the institution. They teach and guide the students to overcome the obstacles and setbacks they may face during the period of their education, including their training period, by collaborating with their mentors. They enable mentees to work out creative solutions with help from the mentors through the dissemination of their knowledge and experience, while also teaching the mentees how to adopt a flexible attitude towards learning and how to cope with diverse situations. Other significantly valuable benefits of mentoring programs for students include better academic performance, increased research productivity, skills development, and career satisfaction. In addition to a sense of personal gratification, students develop self-efficacy and imbibe generic skills that incorporate a set of life and work skills [1, 5].

To experience the positive outcomes of mentoring programs, a successful relationship between the mentor and his mentee is required; it is a relationship that is built upon trust and confidentiality with a clear set of boundaries in terms of its limits, restrictions, and adequate duration [2, 6]. In addition, this mentoring relationship can be an enduring process that supervises the student from his/her first year attending university to his/her last years of study [4].

There are different approaches for mentoring programs, and each approach has unique central goals. One such approach is one-on-one mentoring. The main goal of this approach is to provide personal meetings between the mentor and a single mentee and to establish a strong relationship between the mentor and mentee [6].

Another approach is group mentoring. This is usually used when the mentor would not be able to provide the same environment as in a one-on-one mentoring program and the aim of this approach is different [7]. The aim of group mentoring is to promote positive peer cooperation, enhance group-work skills, and help the mentees to improve their academic performance and overall grades [8]. Regardless of the variety of benefits behind mentoring programs, there are still universities around the world that lack mentoring programs for medical students [9].

At Sultan Qaboos University (SQU), the medical degree is divided into pre-clinical and clinical, and the pre-clinical is further divided into Phase I and Phase II. Both phases have a mentoring program that has been utilized as a part of the integrated learning curriculum. The aim of this cross-sectional study was to assess the mentoring program available for Phase I and Phase II medical students in the College of Medicine at SQU and to investigate the correlation, if any, between the mentoring program and the academic performance of students. This study is necessary to assess the effectiveness of the mentoring program on the academic performance of the students as defined by their cumulative grade point average (CGPA). 

## Materials and methods

Study design

A comparative cross-sectional study was conducted to assess the correlation between the involvement of medical students in the mentoring program and their overall academic performance (represented by students’ CGPA). 

Study site and participants

The study participants included randomly selected medical students in Phase I (n = 74) and Phase II (n = 46) in the College of Medicine at SQU, Sultanate of Oman. The data were collected from the students through an online questionnaire from October 2020 to December 2020. 

Sample size

The number of medical students in Phase I and Phase II who were enrolled in the study was 120. The sample size of the participants was calculated by the following formula: SS =Z2 * (p) * (1-p) / C2 in which SS is the sample study; Z is the Z value (to be specific, 1.96 for 95% confidence); p is the percentage picking a choice which will be expressed as a decimal, and 0.5 and C representing the confidence interval that is 8.77 [10]. The number of participants was increased beyond the minimal sample size calculated to avoid the miscalculation that might result from the study dropout rate.

Sampling method and study tools

To reach the aim of this study, an online questionnaire was delivered to Phase I and Phase II medical students at the College of Medicine, SQU. One hundred and twenty (120) participants were selected by simple random sampling using computer-generated numbers. The approved questionnaire was distributed among the students. The questionnaire included a total of 10 straightforward questions. An explanation of the aim of the study and its objective was provided. The questionnaire included the demographic data of the students, such as gender, phase, and cohort. To achieve the study aim, the questionnaire included the frequency of meetings between the mentee and the mentor per semester and the CGPA of the mentee. Students who were not involved in any mentoring programs were used as a comparative group. Before the dissemination of the questionnaire to the students, the validity and reliability (reflection of consistency) of the questionnaire was measured. The validity of the questionnaire was ensured by considering the following measures: 1) enhancing the awareness of the questionnaire between the students in order to collect the required data and 2) running a pilot study before the actual conduction of the questionnaire to get feedback, which was used to improve the quality of the questionnaire [11]. The reliability of the questionnaire was measured by its internal consistency (the Cronbach’s alpha coefficient > 0.7) [12]. Therefore, the questionnaire used in the cross-sectional study was highly reliable. 

Ethical approval

Informed consent for the study participants was included in the questionnaire during submission. Moreover, the demographic data, the responses of the participants, and their confidentiality were maintained throughout the study. Ethical clearance of the study protocol was approved by the Medical Research Committee at Sultan Qaboos University in October 2020 (MREC #2313).

Data analysis

All the collected data were recorded and analyzed. The differences between both groups (actively involved in mentoring and those who were not involved in any mentoring program) were evaluated by the student’s t-test for statistical significance. One sample student-t-test was used to compare the mean; a value of 2.5 was used. Mean values greater than 2.5 were considered agreeing to statement while values lower than 2.5 were considered as disagreeing. A p-value < 0.05 was considered significant. The test used for the statistical analysis for the association between the two variables was the Pearson rank correlation analysis test. The effect size was calculated using Cohen’s equation: d = mean1 (gp1) - mean2 (gp2)/avg standard deviation (SD), where the avg SD is the average of both standard deviations. Cohen’s d of 0 to 0.2 standard deviations means small effect, 0.2 to 0.5 means medium effects, and > 0.5 means large effects [13]. The description of the analyzed data was presented by using different statistical methods, along with data and graphical representation tools of Microsoft Excel (Microsoft® Corp., Redmond, WA), such as a table, pie chart, and scatter plot to demonstrate the correlation.

## Results

To achieve the goal of this cross-sectional study, the questionnaire was delivered electronically to Phase I and Phase II students. One hundred responses were received, 50 male and 50 female responses (response rate: 83.3%). There was equal participation in the questionnaire of 50 males and 50 females. Most of the participants were Phase II students from cohort 2019 at 70% (n = 70) and 30% (n = 30) of Phase I students from cohort 2018.

**Figure 1:**
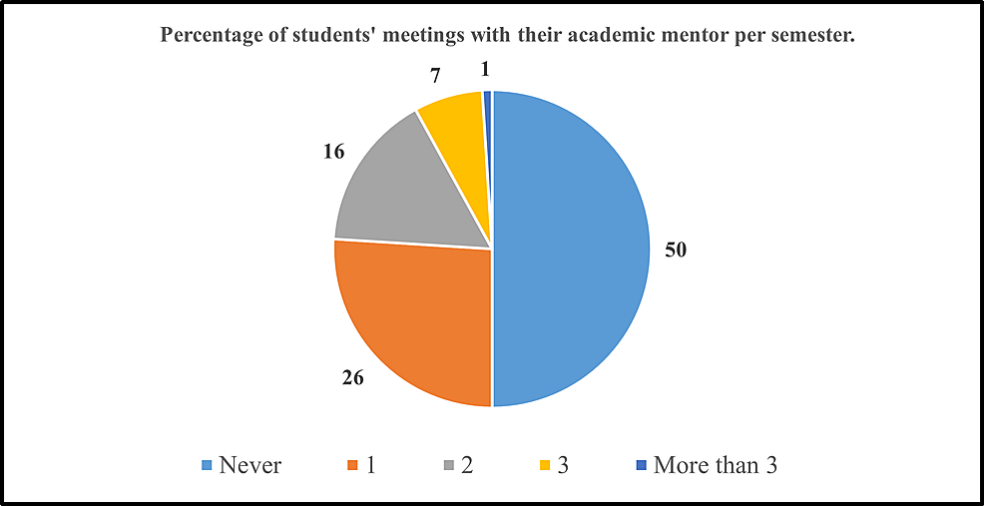
Percentage of students’ meetings with their academic mentor per semester

Figure 1 shows the frequency of the meetings (expressed as a percentage) arranged between the student with his or her academic mentor per semester. Interestingly, the results established that exactly half of the number of the overall respondents have never met with their mentors. However, the other half of the respondents were involved in the mentoring program and used the opportunity to arrange meetings with the mentor for at least one visit per semester. The number of virtual meetings between mentors and mentees during the online study period due to the pandemic conditions was also recorded.

Fifty percent of the respondents (50 students) have never met with their mentor. The other 50% of respondents who met with their mentors were divided into many sections as follows: 26% of the students met with the mentors once in a semester, 16% of the students met with the mentors twice in a semester, 7% met three times with their mentor, and only 1% which corresponds to only one student met with his or her mentor more than three times in a semester. Comparing the average CGPA for both groups of students (those who were actively participating in the mentoring program and those who never met with their mentors) showed that there was no meaningful difference between both groups (Cohen’s d value = 0.24).

Those who were actively involved in the mentoring program (50% of respondents) showed positive response rates regarding the effectiveness and value of the mentoring program (rates exceeded 78%). A strong positive agreement was displayed by this group of participants for items describing that mentoring helped to achieve and set study and career goals effectively and that it was worth the time spent participating in it (82%). There was a strong positive agreement for the item that students would continue to collaborate with their mentors for the next ongoing years of their academic study (87%). In this group, a moderate agreement was evident for the item that without the help of my mentor, the GPA would have been lower. Table 1 shows the survey results, to detect significance, a comparison of means was performed. A test value of 2.5 was used for comparison. Survey statements with a mean value greater than 2.5 were considered as agreeing to question, while those less than 2.5 were considered as disagreeing. The null hypothesis (H0) was rejected because, as inferred from statistical analysis, the t-statistics (7.01) were greater than the t-critical value (2.01); thus, the results were significant.

**Table 1:** Students’ Perception Towards the Mentoring Program Likert scale rating is used to detect the number of respondents under each category: 4: strongly agree; 3: agree; 2: disagree; 1: strongly disagree * P-value is derived from comparison with a hypothetical test value of 2.5 using one sample student-t-test. GPA: grade point average

Items	Strongly agree	Agree	Disagree	Strongly disagree	Mean	* P-value
1. I have set clear goals and objectives with my mentor.	78	12	6	4	3.64	< 0.001
2. My mentor helped me achieve my set of goals and objectives.	81	12	5	2	3.72	
3. My mentor checks my final grades regularly and gives me feedback.	86	10	3	1	3.81	
4. The time I spend with my mentor is effective and productive.	83	9	7	1	3.74	
5. I will continue to collaborate with my mentor for my next years of college.	87	6	4	3	3.77	
6. Without the help of my mentor, my GPA would have been different, lower in a way.	49	5	25	21	2.82	

**Figure 2:**
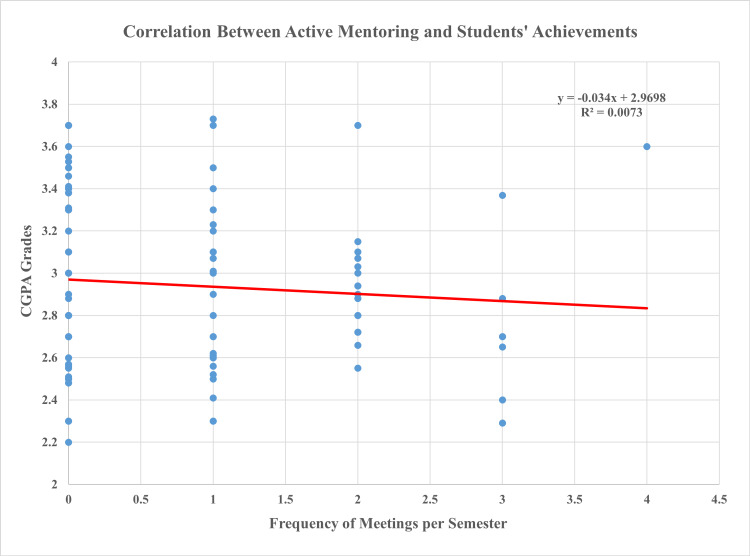
Correlation between active involvement of students in the mentoring program and their academic achievements CGPA: cumulative grade point average

Figure 2 demonstrates the correlation between the active involvement of the students in the mentoring program and their academic overall grades. There was a non-significant correlation between the active enrollment of students in the mentoring program and their academic achievements (R^2^ was 0.007, p-value = 0.757).

## Discussion

Mentoring programs play an essential role in the academic and self-development of mentees, mentors, and institutions, including medical schools [14]. One of the challenges for the success and effectiveness of the mentoring program is how the program is evaluated. Most implemented mentoring programs are evaluated, but there is variability in the quality of their evaluation. Many evaluate the short-term influences and impacts directed over a limited period [1, 15], while others look at the long-term program effectiveness [16]. Long-term surveys are the most commonly used methods for program evaluation though other quantitative and qualitative measures are frequently applied [17- 18].

One tool for evaluating the effectiveness and intervention of a procedure is the Kirkpatrick model [19], which was further amended by Freeth et al. [20]. They demonstrated the four levels of outcome evaluation, including reaction, learning, behavior, and results, where information at each level affected the next one. This model was implemented successfully in the presenting work where we intended to evaluate the effectiveness of the mentoring program implemented at SQU among the medical students for level 1 (students’ reaction towards mentoring), level 2 (new skills learned from mentoring), and level 3 (change in students’ behavior from mentoring). However, for level 4 (institutional impact and change in results), the model was partially applied as we have only evaluated the change in students’ scores (CGPA for only Phase I and Phase II students). However, for terms like "institutional success" measured as an increase in the number of publications, presentations, and awards and the number of higher degrees attained, this was not applicable [21-23]. This may be related to the difficulty in evaluating programs at these higher levels as this requires clear measurable outcomes, alongside more reliable and valid assessment tools, in addition to the time and funding required to do so.

In this comparative cross-sectional study, we aimed to assess the correlation between the involvement of medical students attending SQU in the mentoring program and their academic performance according to their CGPA. Our results indicated that only 50% of the respondents in the study met with their mentors at least one time each semester, and there was a non-significant correlation between the enrollment into the mentoring program and the performance of the student (R^2^ is 0.007, p-value = 0.757). Furthermore, both groups of students, whether they were involved in the mentoring program or not, still achieved high-performance grades as evidenced by a high CGPA. This reveals that there is no direct relationship between the academic achievements of students and their enrollment into the mentoring program. 

Among 120 participants in the study, 100 responses were received, 50 male and 50 female. There were no gender differences in terms of awareness and perception toward the mentoring program among participants, and gender differences had no impact on motivating or encouraging students to actively participate in this mentoring program. This concurs with data found in the literature as not enough is known about the impact of male and female attitudes on the mentoring approach, especially in the medical field. Further future research is required concerning this aspect [2].

Moreover, 26% of the students met with their mentors once a semester. A study conducted by Meinel et al. recorded similar results where 14.6% out of 5,843 medical students met with their supervisors one time each semester [24]. This study emphasized the reasons behind the lack of participation of the students in mentoring programs. One of the main reasons was the idea of a mentoring program in the student’s mind in which the student believed that his or her performance would not be affected by the help and guidance from the mentor. Therefore, the outcomes of this study revealed an insufficient correlation as well.

However, another study, designed by Dimitriadis et al. at the Ludwig-Maximilians-Universität (LMU) School of Medicine, Munich, Germany, reported that in a one-on-one mentoring program that was launched among medical students, 308 actively participated in the mentoring program [25]. To further explore the efficacy of the applied program, the study compared the performance (represented by grades) among the participants and non-participants in their secondary-school examinations and Step 1 of the National German Board Examination. Students who choose to participate in the program outperformed those who did not participate. Those participants showed positive responses towards their mentors in terms of career planning, research, providing ideas, and role modeling. The implemented program was perceived by faculty and medical students as satisfying and an effective tool in the professional development of medical students.

From the literature, many reasons were found behind the low participation and high attrition rates of participants in these programs. One of the reasons was the defective training of the educator when taking on the role of mentor. Ramini et al. highlighted this in “Twelve tips for effective mentors” which remains pertinent and relevant [26]. The study mentioned the need to provide mentors with relevant skills and to develop key listening features while also improving their knowledge of professional boundaries before their involvement in mentoring programs. These findings are in accordance with another study conducted by mentors at Faculdade de Medicina da Universidade de São Paulo that emphasized the difficulties surrounding the expectations and activities of mentors [27]. Moreover, low student participation and high attrition rates from mentoring programs can also pose a problem, and this was reported by other studies [26, 28]. This was highlighted as well by a study conducted at the King Abdulaziz University Faculty of Medicine, Saudi Arabia, which found that nearly 60% and 49% of students were attending the group and the one-on-one mentoring programs, respectively. This study concluded that administrative staff motivation and sustained mentorship are required for an effective and successful mentoring program [29].

Furthermore, a major difficulty contributing to the high attrition rates and unsuccessful mentoring appears to be the time constraints for both mentees and mentors who are overwhelmed by many commitments, such as academic and clinical responsibilities [19, 27]. A suggested solution to overcome this structural barrier is to establish a protected time for mentoring to further enhance positive mentee-mentor relationships [15].

Another reason may be poor communication between the mentor and the mentee. According to a study conducted among final year medical students at Great Western Hospital, Swindon, United Kingdom, some students, though recommending the scheme, felt that they did not need a mentor. Others (nearly 20%) chose not to contact their mentors [18]. Nevertheless, finding a mentor in academia represents another challenge for students. In a study conducted by Fricke et al., only 44% of students participating in the program were able to find an academic mentor conveniently [15]. Therefore, it is important to help students to find a suitable mentor and facilitate a good match between mentees and their mentors.

Mentorship has a great impact if it is implemented properly in the modern integrated curricula, and it is considered as an underutilized powerful educational tool [30]. Mentees who participated in successful mentoring programs showed evidence of developing a high degree of professionalism and showed an improvement in their personal growth, along with their knowledge and skills [8].

Study limitations

This study has some limitations. First, medical students are inherently high performers due to their highly selective criteria for admission to a medical college. Therefore, they may not be affected by mentoring and supervision approaches either way, as these students may compensate by their inspiration and motivation to achieve higher grades in their courses. Furthermore, working on a cross-sectional study during the critical period of the COVID-19 pandemic may have added some limitations and restrictions to the study. For instance, the questionnaire of this study was distributed by an online-based survey only. Thus, it was not possible to ensure that the participants were Phase I or Phase II students. Also, despite our interesting results and although the sample study was based on a calculated formula, the sample size is considered a relatively small sample, as well as the fact that our study was carried out in one medical institution only.

## Conclusions

In conclusion, in this cross-sectional study, we received 100 responses of which 50% of the respondents (50 students) had participated in the mentoring program. Accordingly, the correlation between active participation of students in the mentoring program, frequency of meetings with their mentors, and their academic performance represented by CGPA was calculated (R^2^ of 0.007). Our results show a nonsignificant correlation (p-value > 0.05) between students’ enrollment in the mentoring program and their academic achievements, as the CGPA of the students participating was not affected by their active enrollment into the mentoring program. Thus, it is highly recommended to establish further studies regarding the reasons that are preventing the students from getting involved in the mentoring program available at their university on a regular basis. Future studies may further investigate the objectives of the program and the predictable outcomes in both the short and long-term. Furthermore, these studies could assess the effectiveness of the mentoring program in relation to the grades and overall performance of the students by collecting more detailed data about their competency and professionalism. Subsequent studies should also be conducted on a wider scale that may involve other medical colleges to find an acceptable degree of variability among participants.
